# Quantification of abdominal aortic calcification: Inherent measurement errors in current computed tomography imaging

**DOI:** 10.1371/journal.pone.0193419

**Published:** 2018-02-28

**Authors:** Ruben V. C. Buijs, Eva L. Leemans, Marcel Greuter, Ignace F. J. Tielliu, Clark J. Zeebregts, Tineke P. Willems

**Affiliations:** 1 Department of Surgery (Division of Vascular Surgery), University Medical Center Groningen, University of Groningen, Groningen, The Netherlands; 2 Department of Biomechanical Engineering and Physics, Academic Medical Center, Amsterdam, The Netherlands; 3 Department of Radiology, Academic Medical Center, Amsterdam, the Netherlands; 4 Department of Radiology, University Medical Center Groningen, University of Groningen, Groningen, The Netherlands; Nagoya University, JAPAN

## Abstract

**Objective:**

Quantification software for coronary calcification is often used to measure abdominal aortic calcification on computed tomography (CT) images. However, there is no evidence substantiating the reliability and accuracy of these tools in this setting. Differences in coronary and abdominal CT acquisition and presence of intravascular contrast may affect the results of these tools. Therefore, this study investigates the effects of CT acquisition parameters and iodine contrast on automated quantification of aortic calcium on CT.

**Methods:**

Calcium scores, provided in volume and mass, were assessed by automated calcium quantification software on CT scans. First, differences in calcium scores between the abdominal and coronary CT scanning protocols were assessed by imaging a thorax phantom containing calcifications of 9 metrical variations. Second, aortic calcification was quantified in 50 unenhanced and contrast-enhanced clinical abdominal CT scans at a calcification threshold of 299 Hounsfield Units (HU). Also, the lowest possible HU threshold for calcifications was calculated per individual patient and compared to a 130 HU threshold between contrast-enhanced and unenhanced CT images, respectively.

**Results:**

No significant differences in volume and mass scores between the abdominal and the coronary CT protocol were found. However, volume and mass of all calcifications were overestimated compared to the physical volume and mass (volume range: 0–649%; mass range: 0–2619%). In comparing unenhanced versus contrast-enhanced CT images showed significant volume differences for both thresholds, as well as for mass differences for the 130 vs patient-specific threshold (230 ± 22.6 HU).

**Conclusion:**

Calcification scoring on CT angiography tends to grossly overestimate volume and mass suggesting a low accuracy and reliability. These are reduced further by interference of intravascular contrast. Future studies applying calcium quantification tools on CT angiography imaging should acknowledge these issues and apply corrective measures to ensure the validity of their outcomes.

## Introduction

Much like calcification of the coronary wall, abdominal aortic calcification has garnered interest for becoming an important independent risk factor for cardiovascular health [1–4]. Since vascular calcification is a proxy measure for a prolonged disease state of the medial or intimal wall, it seems reasonable to investigate clinical outcomes based on calcification measurements [5–6]. It is a diagnostic feature, most often visualized with computed tomography (CT) scanning. Additionally, it has been associated with other endpoints as well, such as renal disease, non-alcoholic fatty liver disease and colorectal anastomotic leakage [7–9]. The conclusions of these studies are directly linked to the reliability of the method of measuring the extent of aortic calcification. However, there is no scientific evidence substantiating the accuracy and reliable use of currently available automated measurement methods for aortic calcification.

As many of the aortic calcification measurement methods are based on studies performed on coronary arteries, the assumption of equal accuracy and reliability with aortic measurements may be unfounded. There are important differences that should be accounted for, such as vessel size and wall thickness, hemodynamics and the presence of different surrounding organs. The use of intravenous contrast, which is commonplace in abdominal CT imaging, contributes also to difficulties in measuring calcification. The presence of contrast can overshadow calcified structures of similar or lower radiopacity and therefore interfere with the reliable measurement of calcified entities [10].

The scoring tools for coronary artery calcifications should only be applied on CT scans obtained with a coronary CT protocol or equivalent type protocols, without the use of intravenous contrast [11]. This study was performed to assess the effects of CT acquisition parameters and iodine contrast on the measurements of aortic calcification on CT images. The results provide new insights on the low reliability of calcium quantification on CT, under these different circumstances.

## Methods

### Study design

To evaluate the influence of CT acquisition parameters and iodine contrast on the quantification of aortic calcification, this study was split in two parts. Part one consisted of multiple CT scans of a validated thoracic phantom under two scanning protocols, coronary and abdominal. As the phantom contains calcium elements with known mass and volume, this part allows for the evaluation of the effects of scanning protocols on calcium quantification results. Part two of this study focuses on the quantification of calcified vessel elements in unenhanced versus contrast-enhanced, retrospectively collected, clinical abdominal CT scans. The Institutional Ethical Review Board (METc 201600621) waived the requirement for informed consent and approved this study. All data were anonymized after collection.

### Phantom data acquisition

**Fig 1:**
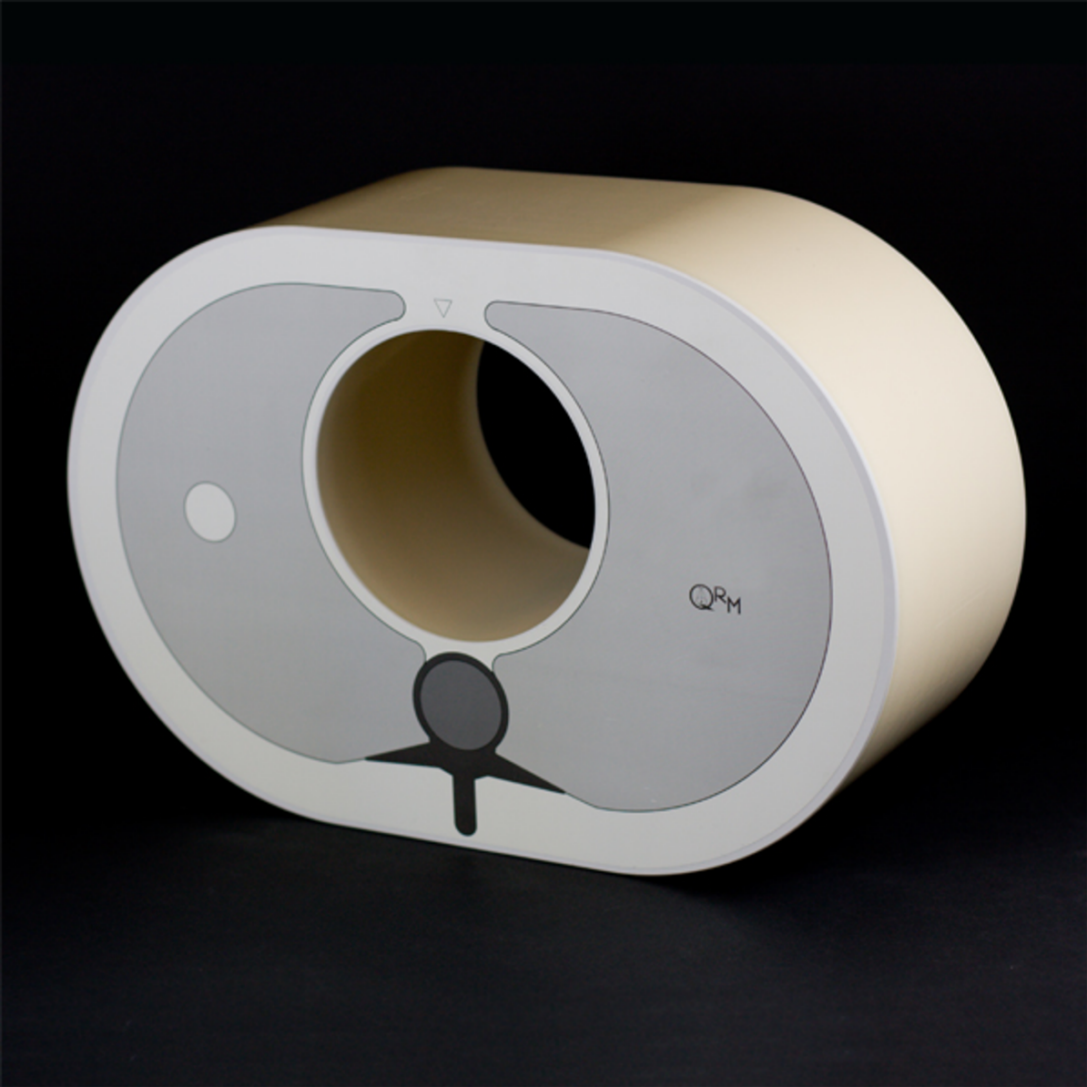
The anthropomorphic thorax phantom. (Reference image provided by QRM GmbH).

**Fig 2:**
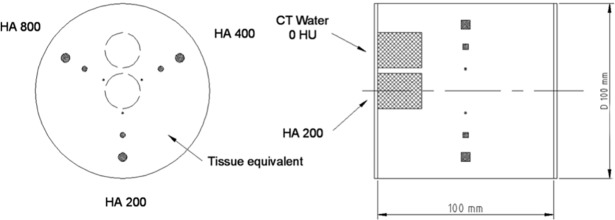
The cardiac calcification insert. Frontal (left) and sagittal (right) view. (Reference image provided by QRM GmbH). It contains nine cylindrical calcifications with different masses and volumes, as well as one large water equivalent element and one large calcium hydroxyapatite calibration element.

Two standardized and clinical protocols for the coronary arteries and the abdominal aorta, respectively, were compared in a phantom study. A validated thorax phantom (Fig 1, QRM-Thorax, QRM GmbH, Moehrendorf, Germany) was used for coronary calcification scoring. The phantom consists of an anthropomorphic thorax of tissue equivalent material with a removable cardiac calcification insert [12–14]. The insert contained nine cylindrical elements (Fig 2) organized in three series of different calcium hydroxyapatite densities and sizes (Table 1).

**Table 1:** Cardiac calcification inserts characteristics.

Spot	Volume (mm^3^)	Mass (mg)	Density (mg/cm^3^)	Diameter (mm)
**1**	0.8	0.16	200	1
**2**	21.2	4.2	200	3
**3**	98.2	19.6	200	5
**4**	0.8	0.32	400	1
**5**	21.2	8.5	400	3
**6**	98.2	39.3	400	5
**7**	0.8	0.64	800	1
**8**	21.2	17.0	800	3
**9**	98.2	78.6	800	5

All CT images were acquired using a 64-slice CT-scanner (Siemens Somatom Sensation, Siemens Healthcare GmbH, Erlangen, Germany). The phantom was scanned with both a standardized coronary artery and an abdominal aorta protocol (Table 2). Both protocols were identical to the institutional standard for clinical care. Five CT scans of the phantom were made with both protocols. Between each CT scan the phantom was moved 2–5 mm in a random direction to mimic patient movement.

**Table 2:** CT protocol characteristics.

CT protocol	kVp	Effective mAs	Slice thickness (mm)	Collimation (mm)	Convolution kernel	Field of view (mm)	Reconstruction matrix (mm)
Coronary	120	190	2.0	0.6–0.8	b35f	300	512 x 512
Abdominal	120	23–26	2.0	0.6–0.8	b30f	300	512 x 512

kVp: Kilovoltage peak

mAs: milli Ampere seconds

Field of view: Acquisition field of view

### Calcification quantification software

3Mensio structural heart version 6.0 (3Mensio Medical Imaging B.V., Bilthoven, The Netherlands) was used to calculate calcium volume and mass. 3Mensio was built with the intent to not only quantify coronary calcification, but aortic calcification as well. However, the software was only validated for the quantification of coronary calcification [12]. Calcium volume and mass were calculated using a standard threshold of 130 HU [15]. Calcium volume and mass of the small, medium and large calcifications were compared between the coronary and abdominal CT protocols and to the physical volume and mass.

### Unenhanced versus contrast enhanced CT images

To assess the effects of CT iodine contrast on the measurements of aortic calcification, both contrast-enhanced and non-enhanced CT images of the same patients that contained a sufficient length of the abdominal aorta were required. Four-phase liver CT scans allow for these scans to take place in quick succession and were readily available, since these had already been performed as part of routine clinical care. Tri- and bi-phasic liver CT scans were found to a significantly smaller degree. Thus, for the purpose of standardization, only four-phase liver CT scans were included. Consequently, 50 clinical four-phase liver CT scans were retrospectively collected and analysed from all four-phase liver CT scans that were conducted between 2013–2015. The following inclusion criteria were applied to minimize selection bias, whilst maintaining a technically qualitative and representative sample of clinical patients with abundant aortic calcification to quantify. Patients over 65 years of age were included by non-stratified simple random sampling, regardless of the clinical indications or results of the four-phase liver CT scan. Clinical patient endpoints were not collected, as these were not deemed relevant to addressing the main goal of the study. Only patients with complete scan information and data were included. Also, inclusion required a b30f reconstruction kernel at an increment of 2.0 mm. From the four-phase liver CT scans, the first unenhanced scan was used as well as the second contrast-enhanced arterial phase scan. As clinical CT angiography of the abdominal aorta only requires one contrast-enhanced scan, the third (venous) and fourth (late) phase acquisition was not deemed relevant to this study.

**Fig 3:**
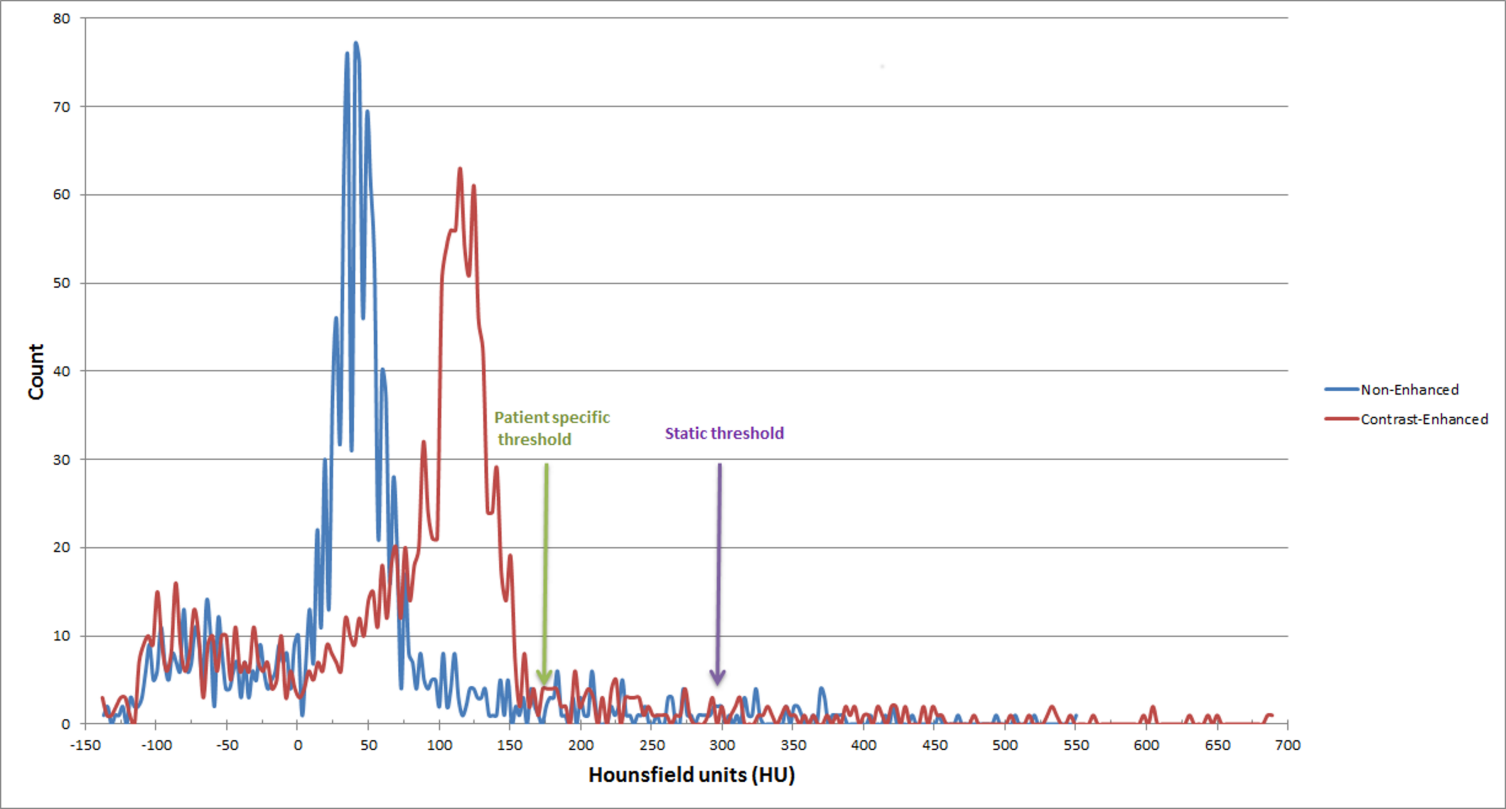
Histogram of the HU levels of the non-enhanced and contrast-enhanced CT scans of one patient. The arrows show the selected thresholds. Purple arrow: static threshold (299 HU). Green arrow: patient specific threshold. HU: Hounsfield Units.

All scans were acquired as part of standard patient care on a Siemens Somatom Sensation 64-slice MDCT-scanner. The collimation slightly differed per patient from 0.6 x 0.6 to 0.8 x 0.8 mm. All arterial phase images were reconstructed to a slice thickness of 2.0 mm, as increasing the slice thickness to the generally applied 3.0 mm would also increase variability in calcification scores [16]. Regions-of-interest were selected on a case-by-case basis for both the unenhanced and the contrast enhanced phase in the abdominal aorta segment found between L1 to L4. In the contrast-enhanced scans, a patient-specific threshold in Hounsfield Units was used to ensure a clear discrimination between contrast and calcifications. This threshold was calculated according to the global thresholding principle (Fig 3). To compare non-enhanced scans and enhanced scans with a similar threshold, a fixed threshold was calculated as well that includes as much calcification signal as possible whilst minimizing contrast signal. This threshold is based on the mean + three standard deviations of the patient-specific threshold. This ensures correct distinction of luminal contrast and calcifications in all scans with calcification signal intensity (Hounsfield Units) within three standard deviations of the mean.

### Statistical analysis

The primary endpoints, in case of non-normal distribution, were reported as summed volumes or mass and their median + range. For normally distributed data, the mean ± SD were provided. Comparisons between protocols were performed using Mann-Whitney U tests. The correlation between calcium scores of non-enhanced and enhanced scans were assessed using related-samples Wilcoxon Signed Rank tests with concordance testing using related-samples Kendall’s coefficient of concordance. Correction for significance was performed according to the Bonferroni method. A post-hoc power analysis was performed for the Wilcoxon Signed Rank tests of the non-enhanced and contrast-enhanced CT scans [17]. No post-hoc power analysis could be performed for the phantom study since Mann-Whitney U tests do not provide z-values, which are necessary to estimate effect sizes for non-normally distributed data. SPSS 20 (Statistical Package for the Social Sciences, IBM, Armonk, NY, USA) was applied for statistical analyses. All data underlying the results of this study were made available for reference.

## Results

### Phantom data results

Results for calcium volume and mass in the coronary and abdominal scanning protocols were compared and tabulated in Table 3. The measurements of the smallest and medium sized calcium cylinder were higher for the abdominal protocol than for the coronary protocol (+2.5% (-100–649) versus -45% (-100–388)), although not significant.

**Table 3:** Percentual differences of the measured volume and mass of the small cylinder, medium-sized and large cylinder with respect to the physical volume and mass in a coronary and abdominal CT protocol.

		Small cylinder difference (%)	Medium cylinder difference (%)	Large cylinder difference (%)
**Volume**	**Coronary**	-45 (0–388)	+66 (98–278)	+56 (104–220)
	**Abdominal**	+2.5 (0–649)	+83 (113–264)	+54 (112–192)
**Mass**	**Coronary**	+131 (0–1563)	+576 (400–1136)	+58 (105–221)
	**Abdominal**	+313 (0–2619)	+648 (462–1076)	+55 (113–193)

The mass measurements of the smallest and medium cylinder were higher, yet not significantly, for the abdominal protocol than for the coronary protocol (+313% (0–2619) versus +131% (0–1563)). Despite the non-significant differences, all measured calcium volumes varied greatly from the true values with the maximum differences ranging up to +649%. For calcium mass measurements the same was true, except for even greater variation of up to +2619%.

### Non-enhanced versus contrast-enhanced CT images

The fixed threshold for this population was calculated to be 299 HU. The mean patient-specific thresholds were 230 ± 22.6 HU. The mean calcium mass and volume measurements, based on the 130 HU threshold, 299 HU threshold and patient-specific threshold, are found in Table 4. The differences between the unenhanced and the contrast-enhanced calcification scores were tabulated here as well. Calcification volume (2421 [16.4–13882] versus 1358 [15.5–12798], p < 0.001) and mass (647 [2.9–6073] versus 583 [5–6630], p < 0.001) measurements were significantly higher for non-enhanced scans in case the 130 HU threshold was compared to the patient-specific threshold. However, in case the fixed threshold was used, the results were higher for contrast-enhanced scans and significantly so for volume measurements (875 [0–9013] versus 1001 [1–11091], p < 0.001). This outlier was found in case calcium mass was measured with the 299 HU threshold in both the unenhanced and the contrast-enhanced scans (442 (0–5251) versus 480 (0.3–6247), respectively). In accordance with the Bonferroni-adjusted significance level of 0,0125, the difference can no longer be considered significant. Since multiple analyses were performed, the effect size measured in this study ranged between 0.2341 and 0.5536, resulting in an overall power of 0.61 to 0.99.

**Table 4:** Results of non-enhanced versus contrast-enhanced CT scans.

	Threshold (HU)	Non-enhanced (median (range))	Contrast-enhanced (median (range))	Difference (median (range))	Wilcoxon signed rank (p-value)	Kendall’s coefficient of concordance (p-value)
**Volume (ml)**	130 vs pat-spec	2421 (16.4–13882)	1358 (15.5–12798)	998.8 (-4665–5280)	< .0001	< .0001
	299 vs 299	875 (0–9013)	1001 (1–11091)	-78.95 (-7217–201)	< .0001	0.02
**Mass (mg)**	130 vs pat-spec	647 (2.9–6073)	583 (5–6630)	90.7 (-3973–870)	< .0001	< .0001
	299 vs 299	442 (0–5251)	480 (0.3–6247)	-11.35 (-4263–443)	0.019	0.024

HU: Hounsfield Units

Pat-spec: patient-specific threshold

Non-enhanced: data of non-enhanced liver CT scans.

Contrast-enhanced: data of contrast-enhanced sliver CT scans.

Difference: difference between non-enhanced and contrast-enhanced CT scans.

## Discussion

This study set out to investigate the effect of technical changes on the measurements of aortic calcification on CT images. The combined results of this study’s tests suggest that the current software technology for aortic calcification measurements of CT images is unreliable. This is due to several important restrictions. These are as follows:

1. The size of the calcification is inversely correlated to the degree of the measurement error; 2. Direct application of software developed for coronary calcification measurements to the abdominal aorta provides grossly erroneous and more importantly, highly variable outcomes; 3. Applying automated calcification measurement software under clinical conditions, i.e. in the presence of intravascular contrast, further disrupts the accuracy of the measurements. The gross overestimation and variability of the calcium measurements is largely caused by partial volume averaging. This is a common artefact in radiography, yet has very little significance in descriptive radiology, since the current generation of CT scanners provide an adequate resolution for visual investigation. Regrettably, voxel-by-voxel analysis by quantification tools, still suffer from partial volume averaging. In short, one voxel on a CT scan can currently contain multiple tissues, like a partial vessel wall, calcifications and blood. The radiodensity of each of these tissues are averaged, since one voxel can only give one HU signal. Few voxels are entirely filled up by small calcifications, yet these do increase the average HU of the surrounding tissues. That explains why smaller sized calcifications are more prone to erroneous measurements than larger ones. This, in turn, creates the illusion that its volume is greater than it in fact is, which is especially pronounced in the case of multiple small calcifications. Partial volume averaging should affect coronary and aortic CT scans equally, therefore its implications may be dismissed to a similar degree. Further detailed research on partial volume averaging in aortic calcification imaging should be performed to substantiate this dismissal. Also, partial volume averaging effects are circumvented to a significant degree in coronary quantification assessment through the application of a semi-quantitative analysis. No such method has been validated for assessing aortic calcification. Additionally, other differences to coronary artery calcification imaging should also be taken into account. For instance, introduction of high-density contrast will create HU signals that overlap with those of any calcified spots and overestimate the volume even further than the partial volume averaging would. Allowing AAA patients to undergo an additional venous phase CT scan could potentially improve the results, and diminish this issue. However, this would entail additional radiation exposure, at least in centres where the arterial phase is deemed most relevant for imaging the aorta in AAA patients. Adequate thresholding and segmentation should minimize these overestimations, although to a limited extent. In fact, especially with threshold-dependent automated quantification tools, it is understandable that major differences in calcification measurements were found between unenhanced and contrast-enhanced CT scans in this study. By choosing the lower HU threshold, the user decides at which minimal HU level an entity on CT is a calcification or not. The 130 HU lower threshold was chosen for unenhanced scans, as it mimics the well-documented use of HU thresholds for coronary artery CT scanning [18]. However, as our results imply, in case intravascular contrast is imaged, this threshold will be too low, resulting in major overestimations of the calcium volume and mass. Therefore, once contrast is introduced, the lower HU threshold for calcification should be raised to correct for the contrast HU level. Since contrast-volume and dispersion of contrast in the patient-dependent volume of blood change between each time a measurement is performed and between each different patient, the threshold should also be patient- and event specific. Previous research by Komen et al. corroborates these results, as the authors compared calcification scores measured with ten different lower HU thresholds for calcification, ranging between 130 and 1000 HU. The authors found that it was not possible to reliably compare calcification scores between scans that were analysed based on different thresholds [10]. Additional research on the correct lower HU threshold for calcification in CT scans of the abdominal aorta is required before any further calcification quantification tools can be applied for predicting clinical outcomes. No measurement tool that has been applied in this field, nor those that are currently in development, are reliable as long as the tools depend on HU values and thresholding as a means of distinguishing between calcified and non-calcified tissues.

This study is first to discuss the technical and practical effects of differing CT scanning settings and the presence of intravascular contrast on aortic calcification scores on clinical CT images. This should be regarded as remarkable considering the studies that have been produced with a variety of presumed aortic calcification scoring tools [7–9, 19]. In these studies, important endpoints, renal health or diabetes, among many others, were correlated to abdominal aortic calcification volume or mass. The studies referenced here are some of many, although an in-depth analysis of these papers would require a dedicated systematic review and meta-analysis. The abovementioned studies proposed correlations between the aortic calcification scores and the respective outcomes. It is therefore paramount that the accuracy of the applied techniques is tried and tested before many of these correlations can be reliably put forward. Especially in light of the questionable reliability of aortic calcification scoring software, as displayed by the wide ranges in this study. This study has several limitations. Firstly, no pre-test power analysis was performed, as no data was available for any suggestions of expected effect size. Therefore, especially the low amount of scans of the phantom study provides a low power. Nonetheless, despite the fact that no firm arguments ought to be made about the difference between the two scanning protocols, this does not negate the obvious discordance between the actual mass and volume, versus what was measured under highly controlled circumstances. Another point of contention could be the fact that the phantom applied in this study is not an abdominal aorta-specific phantom. Therefore, its applicability may be questioned. There is currently no research experience with abdominal aorta phantoms. However, there are publications on the thorax phantom, specifically with regard to coronary calcification [15]. This at least suggests that applying the coronary CT scan protocol on the thorax phantom is reliable, even if the abdominal CT scan protocol is not. Also, this study employed a calcification measurement tool, which has not been clinically validated to reliably measure aortic calcification. This is, however, exactly the reason why this study was performed. Despite the lack of previous applications of the software, the tool did provide adjustability in measuring calcifications in the presence of contrast. Since these software tools work according to similar basic algorithms, the software is assumed to be representative in measuring aortic calcification. Previous research on aortic calcification quantification has generally been aimed at current technology applied in general clinical practice. Therefore, this study only focused on HU-dependent quantification software and 64-slice MDCT technology. However, it should be noted that quantification software packages that do not rely on Hounsfield Units might provide different results. The use of other CT modalities, such as electron beam or dual source CT, is also expected to provide different results to those purported in this study. Lastly, as the patient body type can influence radiation dosage and Hounsfield Unit intensity, prospective collection of this information would have provided additional insights on its potential effects on calcification scores. Yet, as tube voltage and tube current are adjusted based on individual patient body type, these potential confounding effects on calcification scoring will have been minimized in this study.

## Conclusion

Aortic calcification scoring on CT angiography currently suffers from several key issues. The main restriction is the gross, and more importantly, highly variable overestimation of volume and mass measurements. Second, the error increases for small calcium spots, such as those found in the clinical setting. Third, both these issues are worsened by the presence of intravascular contrast. Further research on the reliability of many automated calcification measurement tools ought to be performed before these are further implemented for research and clinical purposes. Experimental studies that rely on the accuracy of these tools without acknowledging the issues purported in this study, should be thoroughly scrutinized before further research is built upon their results.

## Supporting information

S1 File Supporting data of the volume measurements of the phantom CT scans. (SAV)

S2 File Supporting data of the mass measurements of the phantom CT scans. (SAV)

S3 File Supporting data of the four-phase liver CT scan measurements. (SAV)
